# Early detection of Alzheimer’s disease in structural and functional MRI

**DOI:** 10.3389/fmed.2024.1520878

**Published:** 2024-12-12

**Authors:** Rudrani Maity, Vellupillai Mariappan Raja Sankari, Umapathy Snekhalatha, Shubashini Velu, Tahani Jaser Alahmadi, Zaid Ali Alhababi, Hend Khalid Alkahtani

**Affiliations:** ^1^Department of Biomedical Engineering, Faculty of Engineering and Technology, SRM Institute of Science and Technology, Chennai, Tamil Nadu, India; ^2^College of Engineering, Architecture and Fine Arts, Batangas State University, Batangas, Philippines; ^3^MIS Department, Prince Mohammad Bin Fahd University, Khobar, Saudi Arabia; ^4^Department of Information Systems, College of Computer and Information Sciences, Princess Nourah Bint Abdulrahman University, Riyadh, Saudi Arabia; ^5^Riyadh First Health Cluster, Ministry of Health, Riyadh, Saudi Arabia

**Keywords:** Alzheimer’s disease, fMRI, DeepLabV3+, Deep Residual U-NET, feature extraction

## Abstract

**Objectives:**

To implement state-of-the-art deep learning architectures such as Deep-Residual-U-Net and DeepLabV3+ for precise segmentation of hippocampus and ventricles, in functional magnetic resonance imaging (fMRI). Integrate VGG-16 with Random Forest (VGG-16-RF) and VGG-16 with Support Vector Machine (VGG-16-SVM) to enhance the binary classification accuracy of Alzheimer’s disease, comparing their performance against traditional classifiers.

**Method:**

OpenNeuro and Harvard’s Data verse provides Alzheimer’s coronal functional MRI data. Ventricles and hippocampus are segmented using a Deep-Residual-UNet and Deep labV3+ system. The functional features were extracted from each segmented component and classified using SVM, Adaboost, Logistic regression, and VGG 16, DenseNet-169, VGG-16-RF, and VGG-16-SVM classifier.

**Results:**

This research proposes a precise and efficient deep-learning architecture like DeepLab V3+ and Deep Residual U-NET for hippocampus and ventricle segmentation in detection of AD. DeepLab V3+ has produced a good segmentation accuracy of 94.62% with Jaccard co-efficient of 85.5% and dice co-efficient of 84.75%. Among the three ML classifiers used, SVM has provided a good accuracy of 93%. Among some DL techniques, VGG-16-RF classifier has given better accuracy of 96.87%.

**Conclusion:**

The novelty of this work lies in the seamless integration of advanced segmentation techniques with hybrid classifiers, offering a robust and scalable framework for early AD detection. The proposed study demonstrates a significant advancement in the early detection of Alzheimer’s disease by integrating state-of-the-art deep learning models and comprehensive functional connectivity analysis. This early detection capability is crucial for timely intervention and better management of the disease in neurodegenerative disorder diagnostics.

## 1 Introduction

Alzheimer’s disease (AD) gradually impairs memory and cognitive functioning, making daily activities difficult. Although rare, early-onset AD can affect 30–60-year-olds. However, most late-onset Alzheimer’s patients develop symptoms in their mid-60s. AD causes most dementia in those over 65 years (1). Severe cognitive impairment in later stages might result in malnourishment, dehydration, and infections, hence exacerbating existing issues (2). According to the World Health Organization (WHO), the current global prevalence of dementia exceeds 55 million individuals, with a substantial majority, over 60%, residing in low- and middle-income nations. Each year, approximately 10 million new cases are recorded. AD, the most common form of dementia, is believed to be a contributing factor in 60–70% of these cases (3). The predicted estimate of 13.8 million persons by the year 2060 assumes that no progress will be made in medical therapies intended to prevent, decelerate, or treat AD (4). Based on a systematic review of Indian research, dementia prevalence among individuals at the age of 60 and older is estimated at 1.03% (5).

A neurological exam, vitamin B12 blood tests, and a thorough medical and family history evaluation are needed to diagnose AD (6). Homocysteine levels can indicate vitamin B12 deficiency, which can cause neuronal harm through oxidative stress, calcium influx, and apoptosis. Histopathologic evidence from a biopsy or autopsy can confirm Alzheimer (7, 8). Biomarkers for AD diagnosis fall into two groups. PET and CSF studies can assess brain amyloid in the first group. The second group assesses neuronal damage by detecting cerebrospinal fluid tau protein, metabolic activity with FDG, and shrinkage in MRI images (9–11). The effectiveness of MRI in detecting early-stage AD may be compromised when there are minimal structural changes.

Alzheimer’s diagnosis using fMRI offers several benefits: It’s a non-invasive imaging technology that uses no ionizing radiation or intrusive procedures, making repeated tests safe. fMRI measures brain function by measuring blood flow and oxygenation, unlike structural imaging methods like MRI, which indicate AD related brain activity patterns. fMRI can identify brain dysfunction before structural damage, enabling early diagnosis and therapy. It is utilized in AD research to study brain connectivity, identify network failures, and assess therapeutic responses.

Researchers use ventricle size, hippocampus shape, cortical layer thickness, and brain volume to identify AD at an early stage (12). Short-term and long-term memory depend on the medially located hippocampus (13). Neurodegeneration in AD can change the hippocampus’s shape and size. The structural changes in the hippocampus region can be considered an important change in the detection of AD (14).

Hojjati et al. (15) used machine learning to distinguish Mild Cognitive Impairment-Converters (MCI-C) from Non-Converters (NC). They trained and evaluated a support vector machine (SVM) to distinguish MCI-C from MCI-NC with an accuracy rate of 89% for sMRI, 93% for rs-fMRI, and 97% for sMRI with rs-fMRI. Amini and colleagues (16) suggested *k-nearest* Neighbors (*k*-NN), SVM, Decision Trees (DT), Linear Discriminant Analysis (LDA), and Random Forest (RF) for the fMRI identification of Alzheimer’s patients. Multitask feature learning was used to retrieve features. According to the results, the accuracy rates for the *k-*NN, SVM, DT, LDA, RF, and their proposed CNN approach are 77.5, 85.8, 91.7, 79.5, 85.1, and 96.7%, respectively in detection of AD.

Buvaneswari et al. employed the Alzheimer’s Disease Neuroimaging Initiative (ADNI) dataset for AD classification (17). They improved data-driven classification using kernel Support Vector Regression (SVR) by applying kernel-dependent techniques like PCA and t-distributed Stochastic Neighbor Embedding (tSNE). Their kernel-based PCA-SVR technique outperformed with 98.53% accuracy compared to deep neural networks (80.21%) and hippocampus visual features (79.15%).

Mao et al.’s (18) used pre-processed rs-fMRI data and retrieved ALFF (Amplitude of Low-Frequency Fluctuations) and ReHo (Regional homogeneity) parameters. They computed several graph theory-based parameters of the brain functional network. Next, they evaluated several classifiers’ recognition performance and predicted the SVM with the linear kernel as the best classification algorithm. Helaly et al. (19) developed a framework for early detection and classification of AD using deep learning techniques. They analyzed 2D and 3D structural brain images of ADNI dataset using basic CNN architectures. Second, they used VGG19 model for the classification of various stages of AD. They achieved accuracies of 93.61 and 95.17%, for 2D, and 3D multi-class AD categorization, respectively. Following fine-tuning, VGG19 pre-trained model demonstrated remarkable results, attaining better accuracy for AD (97%), EMCI (97%), LMCI (95%), and CN (96%).

Hybrid models are more robust and accurate predictions compared to conventional models. It reduces overfitting and improve performance on unseen data. A hybrid AD diagnostic model might combine traditional neuropsychological assessments with real-time fMRI data and genetic biomarkers, something conventional models cannot dynamically achieve. Hybrid deep learning models like VGG-16-RF (VGG-16 combined with Random Forest) and VGG-16-SVM (VGG-16 combined with Support Vector Machine) can outperform pre-trained models like VGG-16 alone by leveraging the strengths of both deep learning (CNN) architectures and traditional machine learning (ML) classifiers. When RF combined VGG-16, these classifiers use an ensemble of decision trees to aggregate feature decisions, which improves generalization by reducing overfitting. By applying SVM or RF on top of VGG-16’s feature maps, the hybrid models use the deep learning model’s rich features in a more controlled and precise way, leading to improved performance. Combining VGG-16 with SVM or Random Forest allows for more adaptive modeling. The deep features extracted by VGG-16 are processed and further refined by the classifiers, enabling the hybrid models to better adapt to the data distribution and improve performance on specific tasks, such as differentiating between AD and normal subjects.

The scope of the article includes focuses on early diagnosis of AD by analyzing structural and functional changes in the brain using fMRI data. Improving the segmentation accuracy of critical brain regions (hippocampus and ventricles) and enhance the classification accuracy between AD and normal subjects using state-of-the-art deep learning models. The study conducts a comparative analysis of various machine learning and deep learning classifiers to identify the most effective models for AD detection. The proposed work extracts significant functional features from fMRI images and analyzes their potential in distinguishing AD from normal cognitive states.

The objective was to use fMRI data to construct a deep learning system to precisely differentiate the hippocampus and ventricles in AD patients. To identify disease-specific anomalies, the CONN toolset analyses the healthy and Alzheimer’s affected brain functional connectivity. This paper proposes a framework to detect AD patients using unique features from rs-fMRI images. The ALFF and ReHo parameters were retrieved from pre-processed rs-fMRI data, and important brain functional network parameters were estimated using functional connectivity analysis which is done by realignment, slice-timing correction, co-registration, and spatial normalization. A Deep-Residual-U-Net system automates the hippocampus and ventricular segmentation in AD patients. The performance of hybrid deep learning models such as VGG-16-RF and VGG-16-SVM are compared with the machine learning classifiers for AD and normal classification.

Summary of the study’s contribution:

1. This study uses a deep-learning architecture, including Deep-Residual-U-Net and DeepLabV3+ for precise segmentation of ventricles and hippocampus in fMRI images of AD patients.
2. Implements a functional connectivity analysis by extracting functional features like ALFF, ReHo, and various network parameters using the CONN toolbox in MATLAB for robust AD detection.
3. Employs hybrid models like VGG-16 with Random Forest (VGG-16-RF) and VGG-16 with Support Vector Machine (VGG-16-SVM) for binary classification of AD, demonstrating superior performance over traditional classifiers.

The manuscript is structured as follows: Section 1 provides an introduction of the study elaborating the literature review related to the proposed work. Section 2 elaborates on the methodology employed. Section 3 presents the results obtained and offers a detailed discussion of their implications. Finally, Section 4 concludes the study, summarizing the findings and their relevance.

## 2 Methodology

### 2.1 Data collection

The Alzheimer’s fMRI dataset was obtained from Harvard University datasets (20), while the normal fMRI data, which is accessible in coronal view and NIfTI format was obtained from OpenNeuro datasets (21). From these datasets, 80 images, comprising 40 normal and 40 abnormal samples. To ensure a fair analysis, we allocated 30 raw images (15 per class) for testing purposes, while the remaining 50 images (25 per class) were used for training and validation. Additionally, the training and validation dataset underwent a data augmentation process to enhance model performance and generalization. All the subject’s age ranges from 60 to 85 years. The study excluded patients with major intellectual deficits, a history of past serious mental or neurological diseases (apart from AD), and comorbidities involving other underlying pathologies.

### 2.2 Proposed workflow

**Figure 1:**
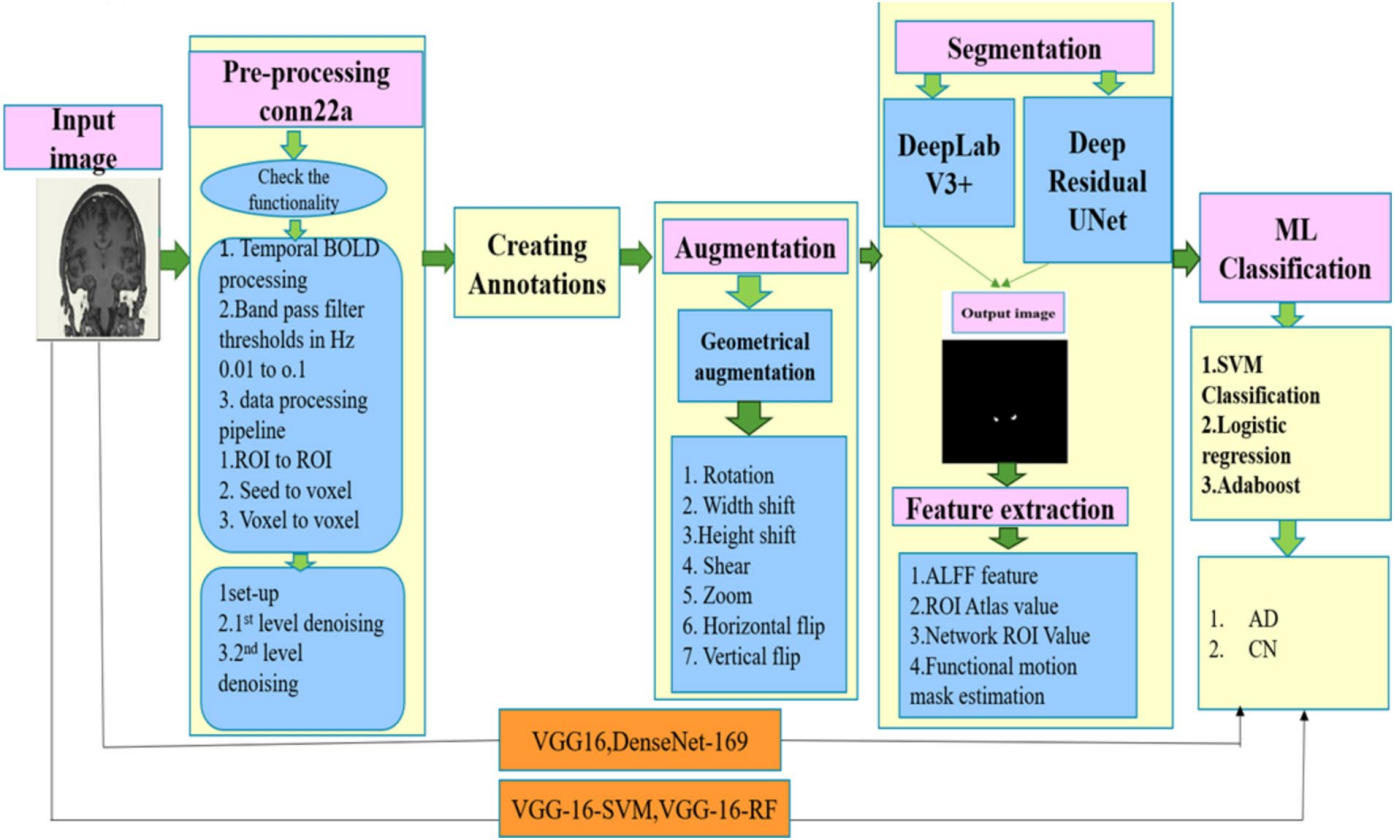
Demonstrate the proposed workflow for classification between normal and AD.

Figure 1 shows the schematic diagram illustrating the overall methods used in the proposed study. At first, raw images were extracted from the dataset and then pre-processed with CONN TOOLBOX in MATLAB to evaluate their functionality using the temporal bold pre-processing technique. In this procedure, both first and second stages of denoising were implemented to complete the pre-processing technique. The extracted pre-processed images are uploaded in Apeer online open-source software to generate the mask images. Then geometrical techniques which included rotation, width shift, height shift, shear, and horizontal flip, was used for data augmentation. Following the data augmentation techniques, segmentation was performed using the DeepLab V3+ and Deep-Residual-U-Net architecture. The functional features such as ALFF features, ROI Atlas value, Network ROI value, and Functional motion mask estimate are extracted from the segmented images for the right and left ventricle as well as the right and left hippocampal regions. SVM, AdaBoost, and logistic regression are three ML classification techniques that leverage the collected features. VGG16 and DenseNet-169 are used for classification of AD and normal. The performance of Hybrid VGG16-RF and VGG16-SVM models were compared with machine learning classifiers.

### 2.3 Pre-processing step

The functional connectivity assessments were performed in AD patients and normal subjects using the CONN TOOLBOX in MATLAB (22). First, the structural and functional images of the same subjects were imported. In the preprocessing phase, functional band-pass filtering was applied, involving the temporal filtering of BOLD data. This process utilized a bandpass filter with cutoff frequencies ranging from 0.01 to 0.1 Hz.

Preprocessing was specifically done in a region of interest (ROI) to examine the connections and interactions between the hippocampus and ventricle. Time series data are collected from these regions, correlations was performed, and functional connectivity analysis was carried out to disclose the functional organization and communication patterns of the brain. The seed-voxel analysis was employed to examine the relationships between activity in the seed area and other brain regions throughout the entire brain. With this connectivity technique, brain areas with similar patterns of activity were discovered and connection maps were generated by computing correlations between each voxel and the seed region. Conversely, voxel-voxel analysis was used to assess the functional connections among individual brain voxels, or three-dimensional pixels. The voxel-voxel approach required calculating correlations between the time series data of all possible combinations of brain voxels, whereas the seed-voxel method focused on the connectivity of a specific brain region (seed). After preprocessing and connection analysis, first-level denoising and analysis were completed, and the connectivity values were evaluated (23).

The preprocessing workflow consists of the subsequent stages:

1. To commence, the structural image should be registered with the corresponding functional brain image.
2. Select temporal processing in the preprocessing configuration, employing a bandpass filter frequency range of 0.01–0.1 Hz.
3. Subsequently, choose the ROI-ROI, Seed-Voxel, and Voxel-Voxel analyses from the menus. The phase is called the “First Level Denoising Process.”
4. Following the completion of the first level of denoising, proceed to the first level of analysis to evaluate the connectivity values.
5. Following this, proceed to the results phase (2nd level Analysis) to assess the hippocampus and ventricle’s oxygen levels and functionality.

### 2.4 Data augmentation

The instance of the Image Data Generator includes augmentation settings to increase the variety of the training data. The parameters included in this study consist of: The rotation range option allows for picture rotation of up to 20 degrees. The parameter “width_shift_range” enables horizontal shifting of images, with a maximum displacement of 10% to the left or right. The height_shift_range, in a similar manner, introduces a vertical shift to the pictures, with a maximum displacement of 10% in either an upward or downward direction. The zoom_range allows for the application of a zoom-in or zoom-out effect with a maximum range of 10%. The shear_range option allows for the application of picture shearing, with a maximum shearing angle of 10%. The parameters “horizontal_flip” and “vertical_flip” randomly flip the inputs horizontally and vertically, respectively. The fill mode refers to the technique used to fill in vacant areas inside the augmented image. The conventional approach involves selecting the pixel value closest to the original image.

The Image Data Generator has generated a cumulative count of 440 augmented images, in conjunction with the initial set of 40 original images for both normal and AD, respectively. After augmentation, the dataset encompasses a grand total of 480 images for both normal and AD.

### 2.5 Deep learning-based segmentation

#### 2.5.1 Segmentation using DeepLab V3+

The DeepLabV3+ architecture (24) is a framework designed for the purpose of semantic image segmentation. The process starts with an input image of dimensions 512 × 512 × 3. The model has a pre-trained ResNet-50 as its encoder, extracting image features from the convolutional layers. The Atrous Spatial Pyramid Pooling (ASPP) module is employed for the purpose of integrating features at several scales. This integration is achieved via the utilization of various operations, including image pooling, 1×1 convolutions, and 3×3 convolutions with distinct dilation rates (namely, 6, 12, and 18). Each of these convolutions is equipped with 256 filters. The process of concatenation involves merging the output of the ASPP module with the features extracted from the preceding encoder layers. Squeeze-and-Excite blocks are utilized to augment the feature recalibration and are implemented both before to and after two successive 3×3 convolution layers, each including 256 filters. The last stage of the process entails the implementation of up-sampling and a 1×1 convolution using a solitary filter. This is then followed by the application of a sigmoid activation function, resulting in the generation of predictions for pixel-wise semantic segmentation. The filter size remains constant at 256 over the whole network, except for a single layer that employs 48 filters. The architectural design of this system efficiently utilizes multi-scale information and feature recalibration approaches to achieve precise semantic segmentation. The main advantage of using DeepLab V3+ is ASPP enables the model to capture contextual information at multiple scales by applying atrous (dilated) convolutions with varying rates. The use of ASPP and decoder refinement allows for sharper and more accurate delineation of object boundaries.

#### 2.5.2 Segmentation by Deep-Residual-U-Net

The ResUNet model (25) has been specifically developed for the purpose of performing image segmentation tasks. The process starts with an input layer that possesses a size of 256 × 256 × 3. The encoder is comprised of five sequential stages, with each stage comprising a stem layer and subsequent residual blocks. The diameters of the filters exhibit a gradual rise, ranging from 16 to 256, therefore facilitating the extraction of features at varying scales. The bridge section improves feature representations by including two convolutional blocks. The decoder employs up sample-merge blocks to reinstate spatial resolution through the process of upscaling feature maps and combining them with skip connections originating from the encoder. The inclusion of both high-level and low-level information in the model is essential for achieving precise segmentation. The last layer employs a 1×1 convolution operation to provide a segmentation mask consisting of a single channel. Additionally, it utilizes a sigmoid activation function to enable pixel-wise binary predictions. The size of the input is aligned with the dimensions of the input layer of the model, whilst the size of the output corresponds to the dimensions of the segmentation mask generated by the final layer. The sizes of the filters used in the convolutional layers are determined by the filter list [16, 32, 64, 128, 256], which is designed to enable multi-scale feature extraction and enhance the effectiveness of image segmentation. The main advantage of using Deep-Residual-U-Net is it incorporates residual blocks, which extract richer and more hierarchical features. This enhances the network’s ability to capture fine details, essential for precise segmentation. Residual connections lead to faster training by optimizing gradient propagation, reducing the likelihood of model degradation. The Jaccard, conformity, and dice coefficients are measured to quantitatively distinguish the Deep-Residual-U-Net segmented image with the ground truth image.

Jaccard co-efficient is measured using the formula as mentioned in Equations 1, 2


(1) $$J\left(P,G\right)=\;|P\cap G|\;|P\cup G|$$

dice co-efficient are calculated using the formula


(2) $$D\left(P,G\right)=2\;|P\cap G|/|\mathrm{P|+|G|}$$

Where ∣P∣ represents Total number of pixels in the prediction; ∣G∣ indicates total number of pixels in the ground truth.

### 2.6 Training and validation

The training process utilized a dataset consisting of fMRI pictures of Alzheimer’s-affected brains, together with corresponding ground truth segmentations. After preprocessing, the brain images and masks of both normal individuals and patients with AD were subjected to random scaling, resulting in dimensions of 1,024 × 1,024 pixels. Initially, there was a collection of 40 images for both normal and AD subjects, respectively. As detailed in Figure 1, we employed seven data augmentation techniques along with one set of raw images, making a total of eight techniques. In our study, this resulted in a dataset of 50 × 8 = 400 images, which was divided into 80% for training and 20% for validation. Additionally, as described in Section 2.1, an independent set of 50 images was utilized exclusively for the testing process.

The training method has 100 epochs, 0.001 initial learning rate, and 0.01 weight decay. In Deep-Residual-U-Net a loss function like binary cross-entropy and an optimizer like Stochastic Gradient Descent (SGD) optimized the model’s parameters during training. In DeepLabV3+, the adam optimizer is integrated with a custom loss function and metrics algorithms for evaluating semantic segmentation. The model is then trained on the training data and utilize the training data for model optimisation and monitoring performance on the validation data.

### 2.7 Feature extraction

The ALFF and ReHo parameters were derived using pre-processed rs-fMRI data. Subsequently, other essential characteristics of the brain’s functional network were computed using graph theory (18, 26).

1. ALFF (Amplitude of Low-Frequency Fluctuations) feature: The ALFF metric measures low-frequency oscillations in fMRI data to measure resting brain activity. A higher ALFF indicates more low-frequency brain activity changes. These changes are intimately connected to brain region interconnection and neuronal activity.
2. ROI Atlas Value is the mean or aggregated functional activity in defined brain areas or ROI. Anatomical or functional brain atlases are used to define these regions, and the studied feature reflects the average degree of activity in each region.
3. The Network ROI Value metric measures brain network or functional connectivity module via functional activity.
4. Functional Motion Mask Process Estimation is used to correct motion-related distortions in fMRI data. Head movement while scanning may cause motion artifacts that complicate the analysis. This capability evaluates and measures movement in fMRI data to reduce its impact on subsequent analytical methods. Four types of characteristics are retrieved independently from each fMRI. Both the Hippocampus and Ventricle portions of the brain undergo the operation separately. The extraction procedure produces four feature values: ALFF, ROI Atlas, Network ROI, and Functional Motion Mask, for both the Hippocampus and Ventricle regions in each image.

### 2.8 Machine learning classification

The supervised machine learning classifiers such as SVM, Adaboost and logistic regression are applied in the proposed study. SVM are utilized for tasks including regression, outlier identification, and linear or nonlinear classification. SVM handles non-linear data effectively by mapping input data into higher dimensions using kernels (e.g., RBF, polynomial). It is useful when the dataset is small and clean.

It provides good performance with well-defined class boundaries. Typically, a linear SVM’s decision function is specified as as given in Equation 3:


(3) $$\mathrm{Sign}\left(w\cdot x+b\right)=f\left(x\right)$$

Where, f(x) determines the judgment function that designates one of the two classes (+1 or −1) for an input feature vector x. If the value included in parenthesis is positive, the sign function returns +1; if it is negative, it returns −1.

x: The data point you wish to categorize is represented by the input feature vector.

w: The weight vector, which establishes the decision boundary’s orientation. It is a vector that has the same size as x.

b: The bias term, which establishes the offset from the origin and the decision boundary’s location. This value is scalar.

Setting SVM classifier hyperparameters: Maximum margin and minimum classification errors are determined by the ‘C’ parameter. Higher ‘C’ values, such as 1, reduce margins and training data errors. The ‘kernel’ option specifies the higher-dimensional data transformation kernel function. A ‘linear’ kernel is used to classify the dataset linearly.

AdaBoost has been found to exhibit a certain degree of resilience in the presence of noisy data. Due to its emphasis on samples that present challenges in classification, this approach can effectively alleviate the influence of noise within the training data. AdaBoost achieves better performance for datasets with smaller feature spaces or misclassification focus. It works well with slightly noisy datasets, ensuring robust performance. The hyperparameters used in the Adaboost are as follows: Number of estimators: 50 and learning rate: 1.

Logistic regression exhibits computational efficiency and possesses the capability to effectively handle voluminous datasets and a substantial number of characteristics without incurring substantial computational burden. Logistic Regression are less prone to overfitting with small datasets. The hyperparameter used in logistic regression are as follows: C = 1; solver = lgbfs. The high level of efficiency exhibited by this technology renders it well-suited for applications that require real-time and online functionality (27). As detailed in Figure 1, we employed seven data augmentation techniques along with one set of raw images, making a total of eight techniques. In our study, this resulted in a dataset of 50 × 8 = 400 images, which was divided into 80% for training and 20% for validation. Additionally, as described in Section 2.1, an independent set of 50 images was utilized exclusively for the testing process. SVM classifier performance is compared to AdaBoost and Logistic Regression classifiers.

### 2.9 Hybrid deep-learning classification

#### 2.9.1 Hybrid VGG16-SVM network

The medical image classification challenge utilizes a pre-trained VGG16 model as a feature extractor (28). The model is initialized using pre-trained weights obtained from the ‘ImageNet’ dataset. The updated VGG16 model is utilized to extract features from the training, validation, and test datasets. Following this, the attributes are utilized as input for a classifier known as SVM. The SVM is set up with predetermined settings, including a linear kernel, reduced regularization, and the activation of probability estimation. The model is trained using the extracted features derived from the training data. Ultimately, the SVM is employed to provide predictions regarding the class labels for the test dataset. The proposed methodology utilizes the transfer learning capabilities of the VGG16 model for extracting features, coupled with the SVM algorithm for classification purposes.

#### 2.9.2 Hybrid VGG16-RF classifier

The features were extracted from a pre-trained VGG-16 model. To generate a feature vector, a predetermined size is assigned to each image. These features may need normalization or scaling based on the RF classifier. RF classifiers accept pre-processed feature vectors. In RF classifier, several hyperparameters are tuned to get the best performance matrices which can evaluate the binary classification. Tree count, maximum depth, and split features are some of the frequent hyperparameters that are used in RF classifiers to get effective result. The parameters such as accuracy, precision, recall, and F1 score were used to assess the RF classifier on a test dataset (29). As detailed in Figure 1, we employed seven data augmentation techniques along with one set of raw images, making a total of eight techniques. In our study, this resulted in a dataset of 50 × 8 = 400 images, which was divided into 80% for training and 20% for validation. Additionally, as described in Section 2.1, an independent set of 50 images was utilized exclusively for the testing process. RF work best with 100 or more trees because they aggregate the predictions of several trees to generate more accurate and robust classifications. Setting n_estimators to 1 creates a single decision tree in our RF model classifier.

## 3 Results

**Figure 2:**
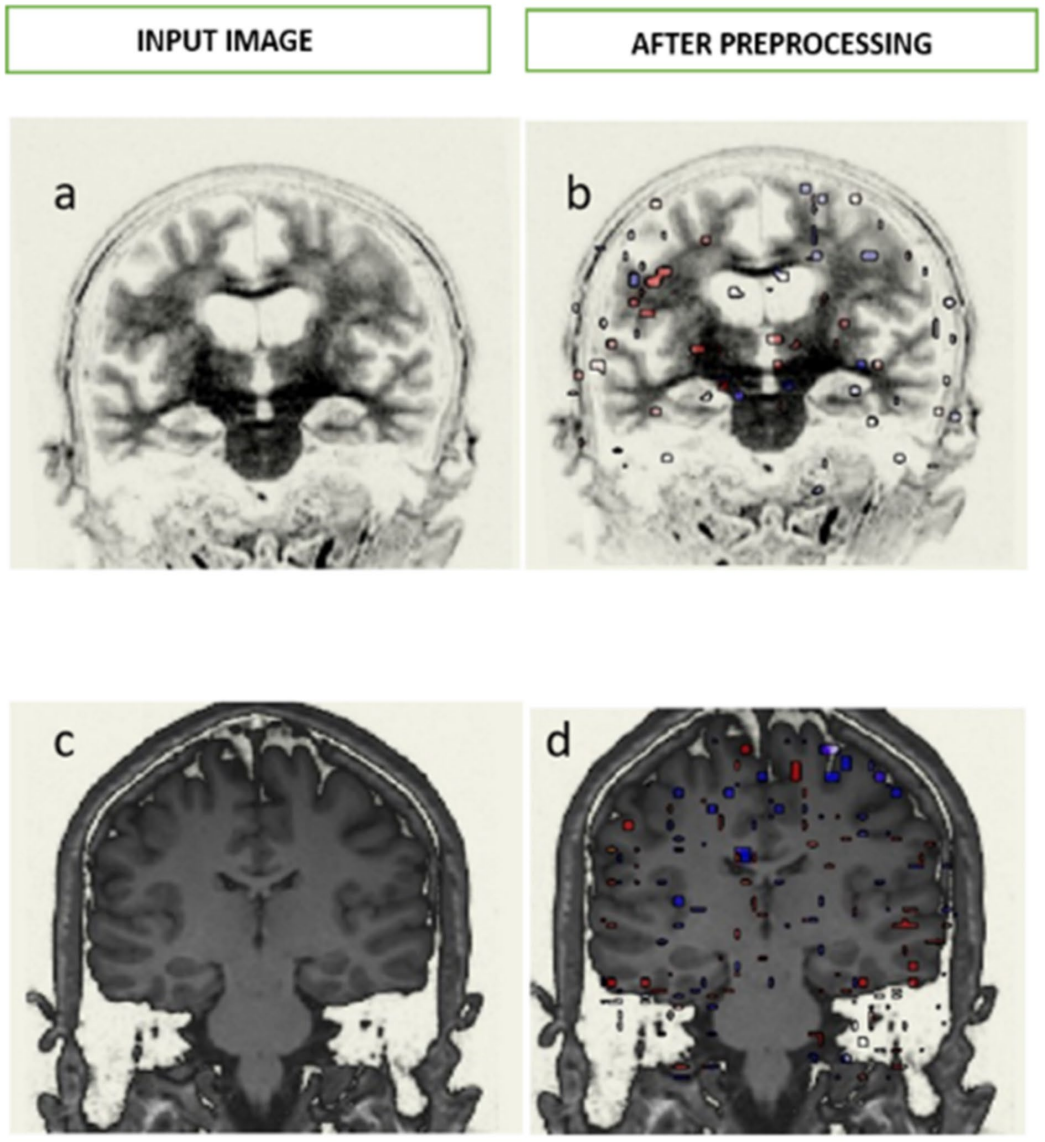
Pre-processing of Alzheimer and normal brain in fMRI images. **(A)** Demonstrates the input image of Alzheimer’s brain and **(B)** demonstrates the output image of Alzheimer’s brain after preprocessing, similarly, **(C)** demonstrates the input image of normal brain and **(D)** demonstrates the output image of normal brain after preprocessing.

Figure 2 demonstrates that a typical brain exhibits elevated BOLD signals in contrast to a brain affected by AD. Within the depicted diagram, the red color is representative of a bold reaction that is positive in nature, whereas the blue color denotes a bold response that is negative in nature. To evaluate the functionality of certain brain areas such as the hippocampus and ventricle regions, the coronal brain scans of participant are aligned both structurally and functionally. The quantification of functional connectivity can be achieved both before and after denoising. Figures 2A,C represents the input image of Alzheimer’s brain and normal brain, respectively, and Figures 2B,D demonstrate the output image of Alzheimer’s brain and normal brain, respectively, after preprocessing.

**Figure 3:**
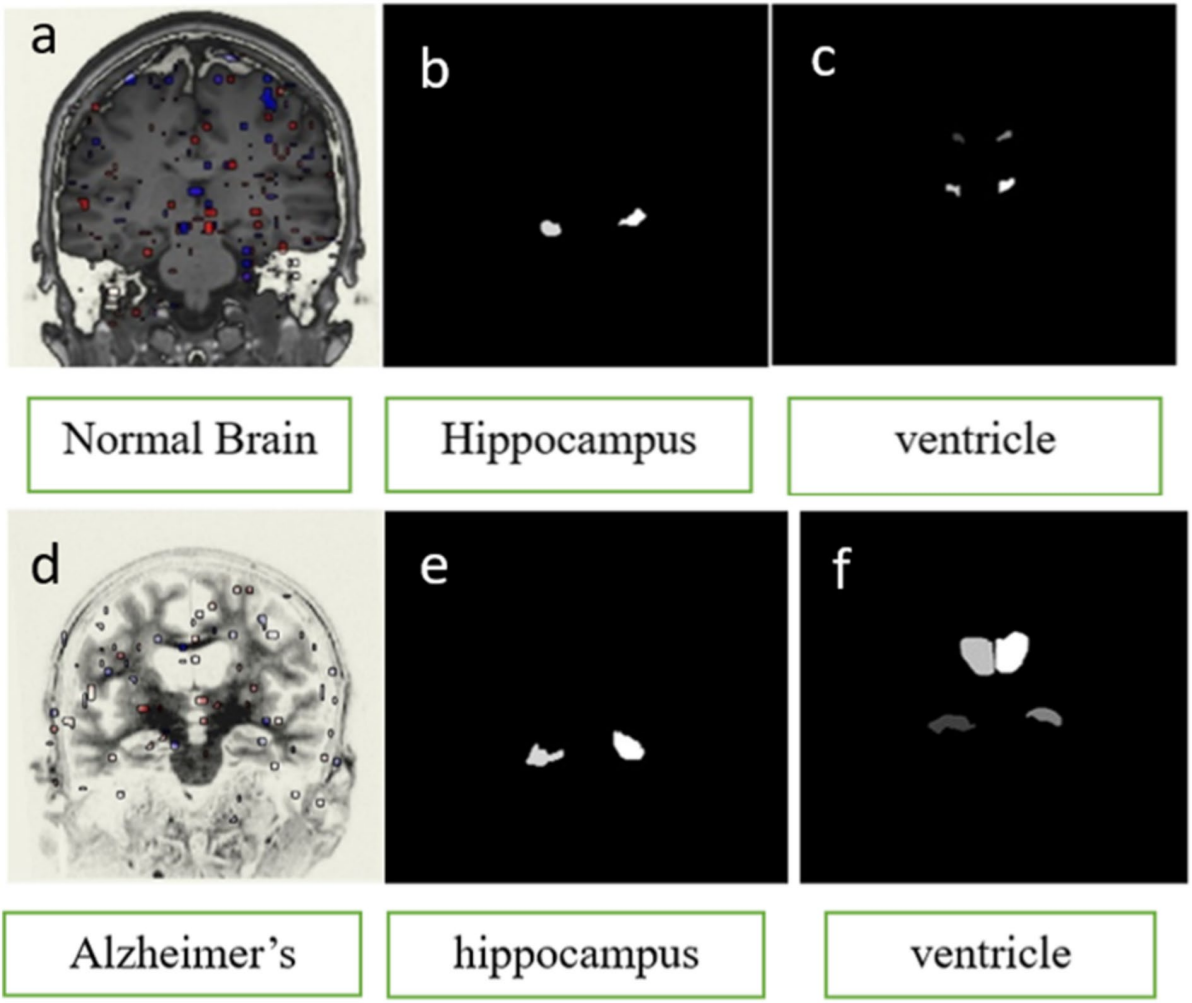
Generation of mask image for segmentation of hippocampus and ventricle region in Normal and Alzheimer’s brain image. **(A)** Input image of NORMAL brain **(B)** mask of hippocampus region in NORMAL **(C)** Mask of ventricle region in NORMAL **(D)** Input image of AD brain **(E)** mask of the hippocampus region in AD **(F)** Mask of ventricle region in AD.

Figure 3 illustrates the process of generating a mask image for the purpose of segmenting the hippocampus and ventricle area in both normal and Alzheimer’s brain images. (3a) The input picture depicts a brain in a normal state. (3b) and (3c) A mask representing the region of the hippocampus and ventricles in a normal brain (3d) The input image depicts a brain affected by AD; (3e) and (3f) A mask representing the region of the hippocampus and ventricles in an AD.

**Figure 4:**
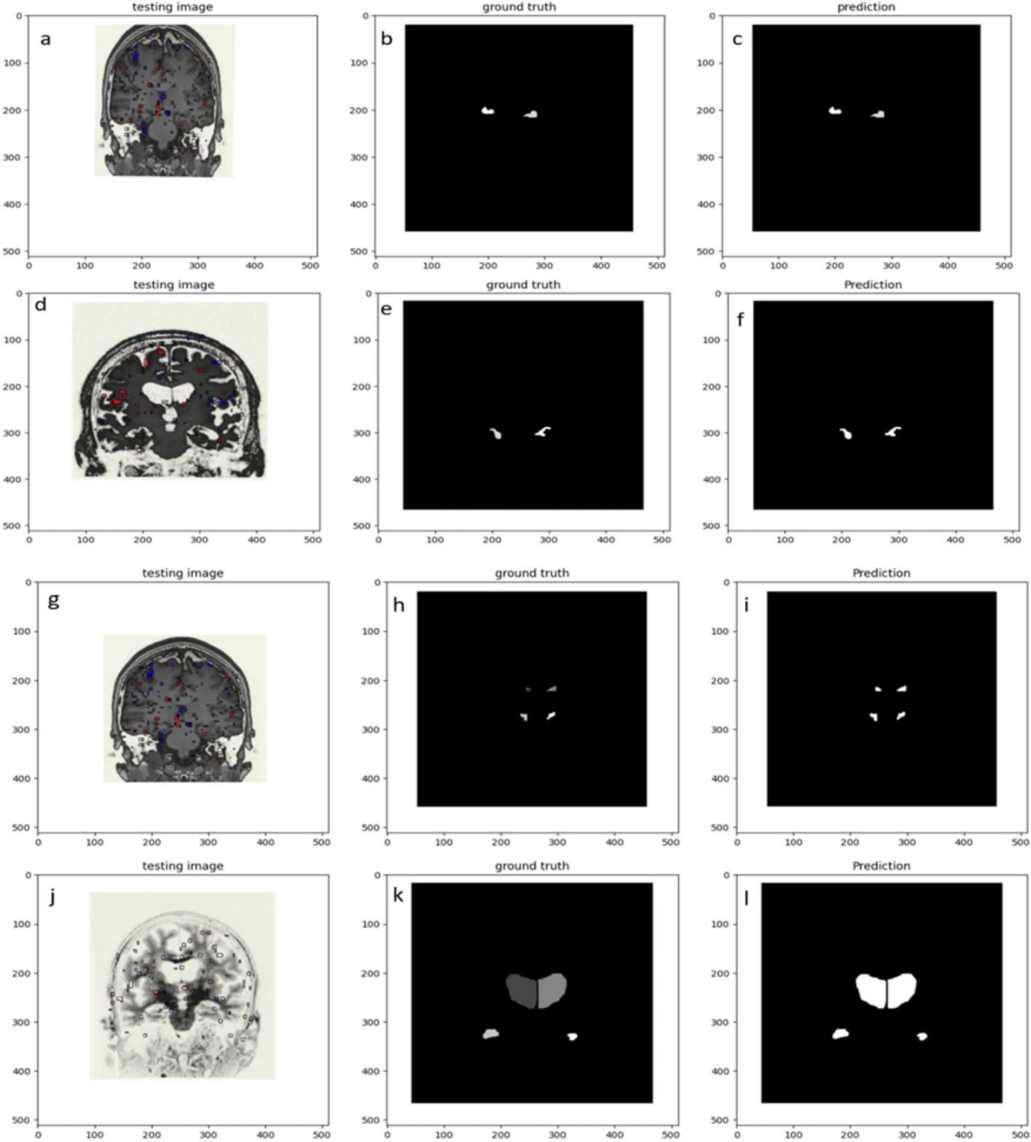
Displays the expected segmented component in healthy people and Alzheimer’s patients. **(A)** Normal brain image; **(B)** Ground truth of hippocampus image in normal; **(C)** Prediction of Hippocampus region in normal; **(D)** Alzheimer’s Brain image; **(E)** Ground truth of hippocampus in AD brain; **(F)** Prediction of hippocampus in AD; **(G)** Normal brain image; **(H)** Ground truth of ventricle region in normal; **(I)** Prediction of ventricle region in NORMAL; **(J)** Alzheimer brain image; **(K)** Ground truth of ventricle in AD; **(L)** Prediction of ventricle in AD.

Figure 4 shows healthy and Alzheimer’s patients predicted segmented components. (4a) and (4g) normal brain image; (4b) and (4h) Ground truth of hippocampus and Ventricle region in normal; (4c) and (4i) Prediction of Hippocampus and ventricle region in NORMAL; (4d) and (4j) Alzheimer’s Brain image; (4e) and (4k) Ground truth of hippocampus and ventricle region in AD; (4f) and (4l) Prediction of hippocampus and ventricle region in AD.

Table 1 depicts the evaluation matrices of normal and Alzheimer’s brain segmentation. In Deep lab V3+ Segmentation method, the segmentation accuracy was obtained as 89.65 and 90.14% in Hippocampus and Ventricle region, respectively. The Jaccard and dice co-efficient calculated in the Hippocampus region of AD was 80 and 79%, respectively. Similarly, in the ventricle region of AD, 82% of Jaccard coefficient and 81% of Dice coefficient were achieved.

**Table 1:** Evaluation matrices of normal and Alzheimer’s brain segmentation.

ROI	Accuracy%	Sensitivity	Specificity	Jaccard coefficient	Dice coefficient
DeepLabV3+					
Normal ventricle	93.21	0.8	0.92	0.82	0.8
Normal hippocampus	91.12	0.78	0.94	0.84	0.85
Alzheimer ventricle	93.74	0.74	0.95	0.85	0.89
Alzheimer hippocampus	89.65	0.81	0.9	0.87	0.82
Deep Residual -UNET					
Normal ventricle	96.29	0.89	0.98	0.83	0.81
Normal hippocampus	95.25	0.86	0.98	0.86	0.88
Alzheimer ventricle	92.85	0.81	0.96	0.84	0.86
Alzheimer hippocampus	94.09	0.82	0.97	0.89	0.84

Deep Residual-UNET has provided the accuracy for segmentation of Hippocampus and Ventricle region of AD as 94.09 and 92.09%, respectively. In the Hippocampus region of AD, the Jaccard coefficient and Dice coefficient are calculated as 89 and 84%, respectively. Additionally, in the ventricle region, the Jaccard coefficient is 84%, and the Dice coefficient is 82%. Deep Residual UNET demonstrated superior segmentation accuracy, achieving 94.09%. The sensitivity and specificity were also noteworthy at 82 and 97%, respectively, surpassing the performance of DeepLabV3+.

Table 2 shows the evaluation matrices of ML classification in AD detection. Among the three ML classifier models, SVM demonstrates the highest accuracy for three types of regions, namely the hippocampus, ventricle, and a combination of features from both regions, achieving accuracies of 90, 92.4, and 93%, respectively. Similarly, SVM exhibits the highest F1-scores across different regions: 90.7% for the hippocampus, 92.7% for the ventricle, and 93.81% for the combination of both the regions.

**Table 2:** Evaluation matrices of ML classification.

Classification type	Features	Performance metrics				
		F1 score%	Precision %	Accuracy %	Specificity %	Sensitivity %
SVM	Hippocampus	90.7	82.9	90	80.4	100
	Ventricles	92.7	86.5	92.4	85.15	100
	Combined	93.81	88.3	93	87.8	100
Adaboost	Hippocampus	90.4	92.39	91	89.81	92.39
	Ventricles	84.81	87.09	85.5	84.1	87.09
	Combined	91.39	96.59	92	88.39	96.59
LR	Hippocampus	58.63	80	68.4	63.88	80
	Ventricles	58.88	89.83	70.4	64.39	89.83
	Combined	58.39	97.56	71.15	64.77	97.56

Table 3 represents the performance matrices of Machine learning, deep learning, and Hybrid models. In this research, three types of machine learning classifications were conducted, including SVM, AdaBoost, and Logistic Regression. Among these, SVM achieved the highest accuracy and F1-score, with values of 91.8 and 92.4%, respectively. Similarly, among all the deep learning and hybrid models, the VGG-16-RF classifier outperformed others, boasting the highest accuracy and F1-score at 96.87 and 96.90%, respectively. Overall, among the machine learning, deep learning, and hybrid models, VGG-16-RF demonstrated superior performance.

**Table 3:** Performance matrices of machine learning, deep learning and hybrid models.

Classifier	F1Score%	Precision%	Sensitivity%	Accuracy%
SVM	92.4	85.9	84.45	91.8
AdaBoost	88.86	92.01	92.83	89.5
Logistic regression	58.63	89.13	94.49	69.98
VGG-16	90.88	89.65	87.11	91.22
DenseNet169	87.02	85.41	83.12	88.54
VGG-16-RF	96.90*	95.91*	97.91*	96.87*
VGG-16-SVM	89.73	82.66	84.54	90.25

Bold font * indicates Excellent performance.

**Figure 5:**
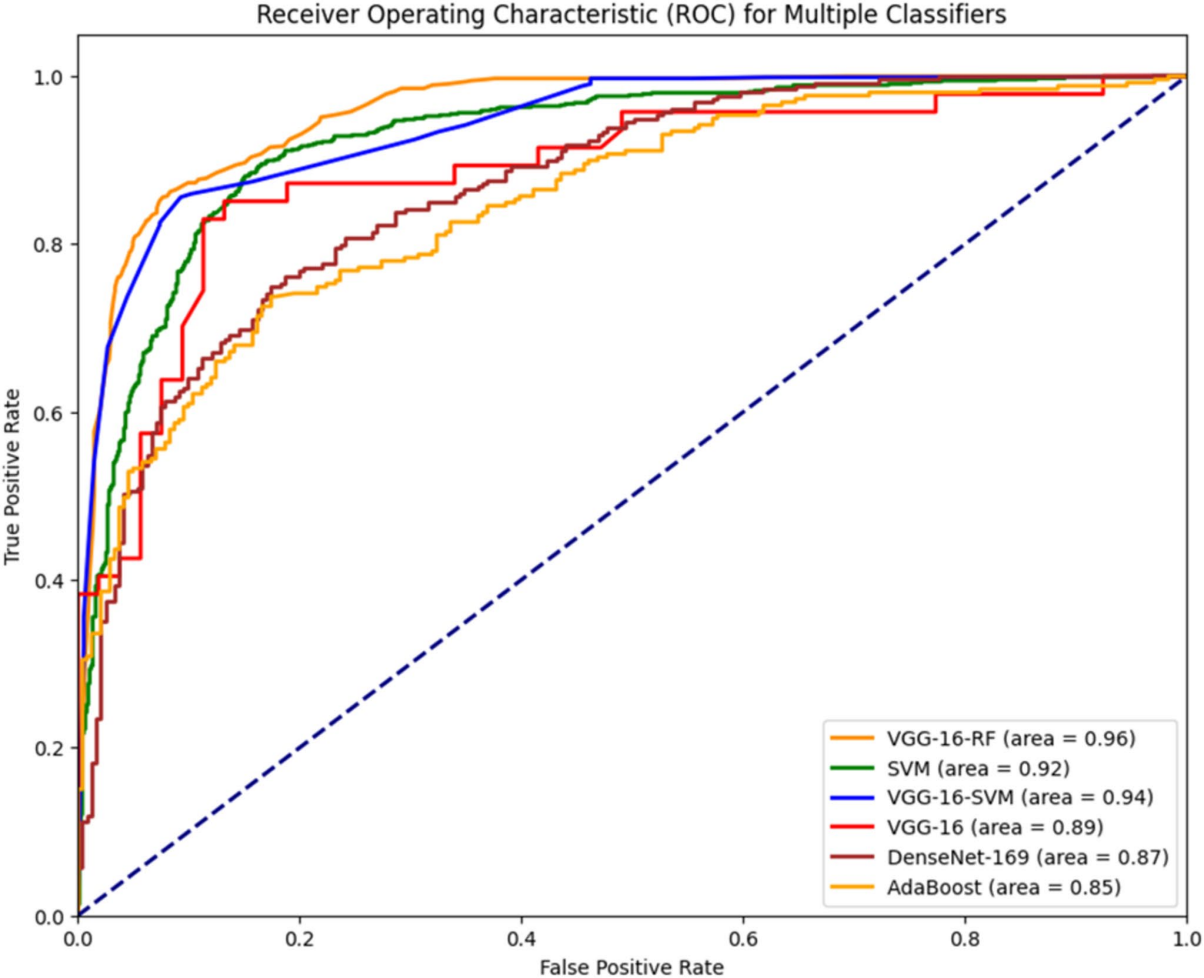
Illustrates the ROC curve for various deep learning and Hybrid models.

In Figure 5, the Receiver Operating Characteristic (ROC) curve visually represents the trade-off between the true positive rate and false positive rate, adjusting the discriminating threshold of a classification model. In the current context, the ROC curve is associated with two classifiers such as VGG16-RF and VGG16-SVM classifier, whose AUC values are 0.96 and 0.94, respectively. VGG-16-RF is a best-performing classifier with the highest AUC. The curve is closest to the top-left corner, indicating excellent sensitivity and specificity. Similarly, the ROC curve for SVM classifier has AUC value of 0.92. The ROC curve illustrates that hybrid models (e.g., VGG-16-RF, VGG-16-SVM) achieve the best classification performance, leveraging both deep learning for feature extraction and machine learning for robust classification. Among the standalone models, VGG-16 and DenseNet-169 also show competitive results, while AdaBoost and traditional SVM are slightly less effective.

## 4 Discussion

The study presents a comprehensive analysis of Alzheimer’s disease (AD) detection and segmentation, highlighting the comparative performance of various models. Functional brain analysis reveals distinct differences in BOLD signal patterns between normal and AD-affected brains, with preprocessing enhancing structural and functional alignment for hippocampus and ventricle evaluation. Deep Residual-UNET outperforms DeepLab V3+ in segmentation tasks, achieving superior accuracy (94.09% for the hippocampus, 92.09% for the ventricle), as well as higher Jaccard and Dice coefficients. It also demonstrates excellent sensitivity (82%) and specificity (97%). For classification, SVM excels among machine learning models, achieving up to 93% accuracy and a 93.81% F1-score when combining hippocampus and ventricle features. Among hybrid models, VGG-16-RF stands out, achieving the highest accuracy (96.87%) and F1-score (96.90%), as well as an AUC of 0.96 in ROC analysis, outperforming VGG-16-SVM (AUC 0.94) and standalone models like VGG-16 and DenseNet-169. The results affirm that hybrid approaches leveraging deep learning for feature extraction and machine learning for classification provide the most robust performance for AD detection and segmentation.

Amini et al. (16) put forward a classification framework for fMRI data that incorporates a range of methodologies, such as *k*-NN, SVM, DT, LDA, and RF classifiers. A CNN architecture was utilized to evaluate the severity of AD through the comparison of fMRI pictures obtained from individuals diagnosed with Alzheimer’s. Regarding the classification outcomes, the CNN model demonstrated a noteworthy accuracy rate of 96.7%. Additionally, it exhibited a precision score of 100% and a sensitivity score of 87.5% for the severe class.

According to the research conducted by Li et al. (30), classifiers have primarily employed two-dimensional (2D) or three-dimensional (3D) images as the primary input data. Although fMRI offers comprehensive 4D data encompassing both spatial and temporal information pertaining to the brain, there is a conspicuous dearth of appropriate techniques for the processing of these 4D images. The VGG 19 model produced an enhanced accuracy rate of 79.21%, whilst the implementation of the ResNet 50 model provided a little lower accuracy rate of 78.70%. The DenseNet 121 model showed a notable increase in accuracy, with a value of 81.58%. Furthermore, the 3D-LSTM model had superior performance compared to the other models, achieved an accuracy of 89.47%.

Sarraf et al. (31) demonstrated the use of fMRI data from normal controls and Alzheimer’s patients using CNN and well-known LeNet-5 architecture, with test data accuracy reaching 96.85%. This experiment indicates that the most effective way to separate clinical from healthy fMRI data by means of extracting shift and scale invariant features and then classify them using deep learning techniques.

Alorf et al. (32) employed rs-fMRI data and deep learning models, specifically Stacked Sparse Autoencoders and Brain Connectivity Graph Convolutional Networks, to classify AD stages and yielded the accuracies of 77.13 and 84.03%, respectively. According to Bamber et al. (33), the OASIS-3 dataset was constructed using a total of 2,168 distinct MRI images. The dataset comprises 1734 training and 434 validation images in which, 20% of the images were used for testing, while the remaining 80 % were allocated for training. The proposed CNN-trained model demonstrated the capability to distinguish between moderate dementia, very mild dementia, non-demented individuals, and mild dementia with a loss-free and 98% accuracy.

In our research, we propose a precise and efficient deep-learning architecture, such as DeepLab V3+ and Deep Residual U-NET, for hippocampus and ventricle segmentation in the detection of AD. DeepLab V3+ exhibits strong performance with a segmentation accuracy of 94.62%, along with Jaccard and Dice coefficients of 85.5 and 84.75%, respectively. Furthermore, among three machine learning classifiers employed, SVM yields promising results with an accuracy of 93%. Notably, the combination of VGG-16 with a RF classifier surpasses other deep learning approaches, achieving a higher accuracy of 96.87%.

The limitation of the study includes as follows: The study primarily relies on a limited dataset sourced from OpenNeuro and Harvard’s Dataverse. This may not adequately represent diverse populations, potentially leading to biases in the findings. Variations in demographics, genetic factors, and disease progression stages across populations are not comprehensively captured, which could limit the model’s applicability to broader contexts. The findings have not been validated on independent, external datasets. This absence of cross-validation or testing on diverse datasets raises concerns about the generalizability and robustness of the proposed framework when applied in real-world scenarios. The study relies on specific tools like the CONN toolbox for functional connectivity analysis, which may not be universally available or easy to use for all researchers.

Some potential areas for future work and research in the field of AD diagnosis using deep learning are as follows:

1. Integrate fMRI data with structural MRI, PET scans, and genetic data. This may improve AD diagnosis and comprehension.
2. Use longitudinal studies to examine brain connection and segmentation changes. This may aid disease progression and biomarker identification for early detection.
3. Validate deep learning models on bigger and more diversified datasets, including medical data, in clinical investigations. This would make the diagnostic procedures more generalizable and reliable (Table 4).

**Table 4:** Performance comparison of the existing literature with the proposed model for AD classification.

References	Input to the model	Model	Accuracy
Amini et al. (16)	fMRI	CNN	96.7%
Li et al. (30)	fMRI	Dense Net 121	89.47%
Sarraf et al. (31)	fMRI	LeNet-5	96.85%
Alorf et al. (32)	rs-fMRI	Stacked Sparse Autoencoders, Graph Convolutional Networks	77.13% 84.03%
Bamber et al. (33)	MRI	CNN Model	98%
Proposed work	fMRI	VGG-16 RF	96.87%

## 5 Conclusion

The proposed study used deep-learning framework for segmenting the hippocampus and ventricles in AD using functional MRI data. By focusing on functional MRI (fMRI) data, the study successfully segments critical brain regions, specifically the hippocampus and ventricles, which are key biomarkers for AD progression. This study suggests a precise and effective deep-learning networks such as DeepLab V3+ and Deep Residual U-NET for the segmentation of the hippocampal and ventricle in the identification of AD. Advanced networks like DeepLab V3+ and Deep Residual U-Net have shown remarkable efficacy, with DeepLab V3+ achieving a segmentation accuracy of 94.62%, along with Jaccard and Dice coefficients of 85.5 and 84.75%, respectively, underscoring its capability in handling complex anatomical structures. Among the three ML classifiers used, SVM has produced good results, with an accuracy of 93%. Furthermore, the hybrid model integrating VGG-16 with Random Forest (RF) delivered the best results among convolutional neural network (CNN) architectures, achieving a highest accuracy of 96.87%, demonstrating the advantages of combining feature extraction strength of CNNs with the decision-making capabilities of ensemble models. The integration of high-performing CNN architectures and hybrid models demonstrates the feasibility of achieving accurate and automated diagnostics. This advancement could lead to better monitoring of disease progression, reduced diagnostic delays, and informed therapeutic strategies. The proposed CNN models play a crucial role in advancing the detection of early AD and potentially influencing the course of the illness through timely intervention.

## Data Availability

The original contributions presented in the study are included in the article/supplementary material, further inquiries can be directed to the corresponding author.
