# Corrigendum: Early detection of Alzheimer's disease in structural and functional MRI

**DOI:** 10.3389/fmed.2025.1589447

**Published:** 2025-03-27

**Authors:** Rudrani Maity, Vellupillai Mariappan Raja Sankari, Umapathy Snekhalatha, Shubashini Velu, Tahani Jaser Alahmadi, Zaid Ali Alhababi, Hend Khalid Alkahtani

**Affiliations:** ^1^Department of Biomedical Engineering, Faculty of Engineering and Technology, SRM Institute of Science and Technology, Chennai, Tamil Nadu, India; ^2^College of Engineering, Architecture and Fine Arts, Batangas State University, Batangas, Philippines; ^3^MIS Department, Prince Mohammad Bin Fahd University, Khobar, Saudi Arabia; ^4^Department of Information Systems, College of Computer and Information Sciences, Princess Nourah Bint Abdulrahman University, Riyadh, Saudi Arabia; ^5^Riyadh First Health Cluster, Ministry of Health, Riyadh, Saudi Arabia

**Keywords:** Alzheimer's disease, fMRI, DeepLabV3+, Deep Residual U-NET, feature extraction

In the published article, there was an error in the Funding statement. It needed to include the same content as provided in the acknowledgment section. The correct Funding statement appears below.

